# Prevalence and Risk Factors of Idiopathic Epiretinal Membranes in Beixinjing Blocks, Shanghai, China

**DOI:** 10.1371/journal.pone.0051445

**Published:** 2012-12-10

**Authors:** Xiao-feng Zhu, Jin-juan Peng, Hai-dong Zou, Jiong Fu, Wei-wei Wang, Xun Xu, Xi Zhang

**Affiliations:** 1 Department of Ophthalmology, Shanghai First People's Hospital, Affiliated Shanghai Jiaotong University, Shanghai, China; 2 Beixinjing Community Health Service Center, Shanghai, China; Zhongshan Ophthalmic Center, China

## Abstract

**Background:**

To determine the prevalence and risk factors associated with idiopathic epiretinal membranes (iERM) in a Chinese population aged 60 years or older in Beixinjing Blocks, Shanghai.

**Methods:**

This population-based study consisted of 3727 participants (89.7% of the eligible). It was performed to describe the prevalence of iERM and possible demographic, systemic, and ocular factors associated with iERM. Each participant underwent a standardized interview and comprehensive ophthalmic examination. iERM was identified and graded from retinal photographs. Then, a case-control study comparing the participants with vs. without iERM was performed to further study the associations between iERM and blood biochemical test results (including fasting plasma glucose, serum creatinine, total cholesterol, and triglyceride), ocular biological parameters (including the axial length, corneal curvature, refractive diopter, intraocular press, and anterior chamber depth), and the data of optical coherence tomography.

**Results:**

The prevalence of iERM was 1.02%. iERM was significantly associated with diabetes (OR: 2.457; 95% CI: 1.137, 5.309) and a higher level of education (OR: 1.48; 95% CI: 1.123, 1.952). Blood biochemical test results and ocular biological parameters showed no significant differences between the iERM and control groups, whereas the incidence of posterior vitreous detachment in the iERM group was much higher than in the control group (26.5% vs. 8.8%), but this difference was not statistically significant. Moreover, the eyes with iERM had poorer visual acuity than the eyes without iERM (P<0.05).

**Conclusions:**

In Beixinjing Blocks, Shanghai, iERM was relatively rare, was associated with diabetes and a higher level of education, and caused a substantial decrease in visual acuity.

## Introduction

Epiretinal membrane (ERM) is a retinal disease resulting in a disturbance of macular vision and predisposing to rhegmatogenous retinal detachment [1], which significantly impair quality of life [2], [3]. ERM is characterized by wrinkling or distortion of the macular surface caused by retinal cell migration and proliferation [4]–[6], and it has been associated with a variety of ocular diseases, such as diabetic retinopathy (DR) [7], [8], retinal vein occlusion [4], [9], retinal detachment [9]–[11], and cataract surgery [4], [5], [7]–[9], [12]. Most cases, however, are termed idiopathic ERM (iERM): they have no antecedent ocular pathology other than posterior vitreous detachment (PVD) or separation [13], [14]. While the pathogenesis of iERM is not fully clear, there is growing evidence that PVD plays a critical role in the pathogenesis of iERM through at least two possible mechanisms [15]. First, transient vitreoretinal traction during the development of PVD may cause dehiscences in the internal limiting membrane (ILM) through which glial cells can migrate and proliferate on the inner retinal surface [16]–[18]. Second, and perhaps more frequently, iERM may result from the proliferation and transdifferentiation of hyalocytes contained within vitreous cortical remnants left on the retinal surface following PVD [19]–[21].

Epidemiological studies contribute to clarify the pathogenesis and risk factors of iERM. Population-based studies [4], [5], [7], [8], [22]–[28] have reported a great discrepancy (2.2% to 18.5%) in the prevalence of ERM among different ethnic groups. It has been suggested that Asians have a lower prevalence of ERM compared with Caucasians [22], [24]–[26]. Similarly, studies [8], [23], [25]–[28] documenting the prevalence of iERM in various populations also shown substantial ethnic variations. The lowest prevalence (3.0%) was reported in the Handan Eye Study [25], and the highest (17.5%) in the Los Angeles Latino eye study (LALES) [23]. Although there have been two population-based studies [24], [25] of the ERM prevalence in China, the differences in study design and methodology resulted in a large discrepancy. The Beijing Eye Study [24], including both rural and urban subjects aged 40 years or older, relied solely on retinal photographs, while the Handan Eye Study [25], including rural subjects aged 30 years or older, relied on retinal photographs and/or optical coherence tomography (OCT). Therefore, further study of ERM in a Chinese urban population is necessary. Moreover, according to population-based studies, in addition to old age [4], [7], [8], [22]–[25], [26], [28], which is a recognized risk factor for iERM, other possible risk factors, such as female gender [26], refractive error [4], [24], [25], [28], diabetes [4], [8], [27], and serum cholesterol [22], are inconsistently associated with iERM. Therefore, in Part I of our study (a population-based study), we aimed to describe the prevalence of iERM in a random sample of the resident population aged 60 years or older in Beixinjing Blocks, Shanghai, China, relied on retinal photographs. Meanwhile we examined risk factors associated with iERM, including ocular, systemic and socio-demographic characteristics.

Previous clinical studies [29]–[31] concerning the risk factors associated with iERM have been susceptible to selection bias because the cases usually came from hospital patients, who were generally at the second or higher stage of iERM and had subjective symptoms. Thus, in Part II of our study (a case-control study), comparing the participants with vs. without iERM from Part I was performed to further study the differences between the two groups. We have studied some possible iERM risk factors in Part I, such as old age, gender and diabetes, so we carried out a blood biochemical test for the cases and controls as a supplementary evaluation. Partial or complete PVD has been found in 80% to 95% of eyes with iERM in large clinical studies [32]–[34], and it is likely that the association between iERM and PVD is universal. Therefore, B-mode ultrasound and OCT examinations were conducted to analyze the existence of PVD in eyes with or without iERM. Furthermore, there is some controversy about the association between refractive error and iERM [8], [23]–[26], so we further examined the axial length, corneal curvature, refractive diopter, anterior chamber depth (ACD), and intraocular press (IOP) in cases and controls.

## Materials and Methods

### Study Design and Population

Beixinjing Blocks, located in the northwest of Shanghai, was selected for the study because of its relatively stable population (43,253 in the 2000 census, and 8,153 aged 60 years or older) and representative demographic and socioeconomic characteristics. Beixinjing Blocks has mostly urban residents whose economic conditions could be classified as middle class in China: average per capita annual incomes among urban households are 36,230 yuan ($5728 US) [35]. Moreover, Beixinjing Blocks has fairly complete health archives for residents and its coverage rate had reached 97.52% in 2001.

This study protocol was approved by the Human Research and Ethics Committee of the Shanghai First People’s Hospital, affiliated Shanghai Jiaotong University, and adhered to the tenets of the Declaration of Helsinki.

#### Part I: Population-based study

Between November 2010 and April 2011, a population-based study of the prevalence of iERM was designed and performed in Beixinjing Blocks. Random cluster sampling was used to select the study sample. Clusters were defined geographically using Residence Administrative Committees (RACs) of approximately 1,000 persons (all ages). By using 2000 census information, the residents of RAC that had total populations larger than 1,500 were subdivided, and the residents of RAC smaller than 500 were grouped in defining clusters for sampling. Forty-two clusters were defined for random sampling. With an estimated 18.9% of the Beixinjing Blocks population aged 60 years or older, the typical cluster was estimated to contain approximately 190 study participants.

The required sample size was calculated based on estimating with 95% confidence the prevalence of ERM in the Handan Eye Study (3.4%) [25]. The required sample size with simple random sampling can be calculated as n≈Z^2^(p)(1-p)/B^2^ where p = 0.034; B = 0.034(0.25) = 0.0085 with a 25% error bound; and Z = 1.96 for a 95% confidence interval (CI). It was assumed that a cluster design effect as high as 1.5 might be present, which would increase the calculated sample size from 1,746 to 2,619. With an estimated response rate of 90%, the required sample size became 2,910. Accordingly, 16 clusters (with an expected study population of 3,040) were chosen by simple random sampling from the list of 42 clusters.

Two days before the examinations, RACs telephoned the eligible residents, informing them of the examination date and the purpose of the study. The field examinations were mainly carried out in the nearest community meeting room, while home inspections were used to collect data of physically disabled residents. Near the end of this study, the eligible residents who had not participated in the field examination on the previously scheduled day were notified by phone about another examination date.

The research group included one lead ophthalmic doctor who had prior experience organizing large-scale epidemiologic studies, four trained ophthalmologists from Shanghai First People's Hospital, affiliated Shanghai Jiaotong University, and two trained physicians from Bingxinjing Community Hospital. Before formal investigation, the members of the research group had trained for two weeks to understand the purpose of the study, methods, and detailed steps for each variable (such as familiar with correct filling sheets, standard operating procedures of inspection equipment, and diagnosis, classification and grading criteria of iERM). Written informed consent was first obtained from all study participants. A detailed interview was conducted to collect information regarding demographics (including age, gender, employment status, years of formal education after kindergarten, height, and weight), histories of diagnosis and treatment relating to systemic comorbidities (such as hypertension, diabetes, and cardio-cerebrovascular diseases) and ocular diseases (such as DR, cataract, and glaucoma). After that, all eligible participants underwent a comprehensive ophthalmic examination. Visual acuity of each eye was measured using the log of the minimum angle of resolution (LogMAR) Early Treatment Diabetic Retinopathy Study (ETDRS) chart at a distance of 4 m, with illumination ≥300 lux. In participants who were wearing glasses in their daily lives, visual acuity was measured with their spectacles. Both types of visual acuity mentioned above are known as the presenting visual acuity [36]. In addition, pinhole-corrected visual acuity was measured in participants with a presenting visual acuity worse than 0.7 in either eye. Anterior segment examinations with a slit-lamp biomicroscope, refractive media and fundus examinations with a direct ophthalmoscope were conducted by the ophthalmologists. If participants’ pinhole-correction visual acuity worse than 0.7 and the vision loss could not be attributed to corneal disease, the examinations were performed after pupil dilatation with 0.5% tropicamide and 0.5% phenylephrine (Mydrin-P; Santen Pharmaceuticals; Japan) except in case of a shallow anterior chamber. A digital 45° non-mydriatic retinal camera (CR-DGi Non-mydriatic Retinal Camera; Canon Inc., Tokyo, Japan) was used to obtain color retinal photographs of ETDRS standard field 1 [37] (centered on the optic disc) and field 2 (centered on the macula) for each eye.

The retinal photographs were assessed respectively by two ophthalmologists with retinal subspecialty training for the presence of ERM and its grading. The prevalence-adjusted bias-adjusted kappa statistic was 0.82 for the presence of ERM, and the kappa statistic was 0.70 for its grading. In case of doubt, the lead ophthalmic doctor reassessed the retinal photographs to make the final diagnosis and grading. In our study, ERM was subdivided as idiopathic or secondary. iERM was defined as ERM occurring in eyes without a secondary cause, such as DR (at least a history of diabetes associated with retinal microaneurysms), retinal vascular disease, retinal detachment, or history of cataract surgery. Moreover, iERM was graded using the method described by Klein et al. [7], which divides iERM into two types, cellophane macular reflex (CMR) and premacular fibrosis (PMF). CMR was defined as a patch or irregular, increased reflection from the inner retinal surface. PMF, a more severe type, was defined as a grayish and opaque appearance of the inner retinal surface combined with superficial retinal folds or traction lines. Participants with both CMR and PMF were allocated to the PMF group. Thus, iERM was detected in 34 participants (1.02%), who were all later confirmed by OCT.

#### Part II: Case-control study

In November 2011, the 34 participants with iERM as the case group and 34 healthy participants randomly selected (using a computer-generated random number table) as the control group from the participants without ERM were further examined in the Beixinjing community health service center. Cases and controls were well matched in age, sex, body mass index (BMI), and the iERM-associated risk factors (diabetes and higher level of education) obtained from Part I. After explaining the purpose of this study, we obtained the written informed consent from all participants.

Two ophthalmologists, two optometrists and one retinal specialist performed the following examinations. Blood samples were collected for testing plasma glucose, serum total cholesterol, creatinine, and triglyceride after an overnight fast. The uncorrected distance visual acuity (UCDVA) was measured using the ETDRS chart as described in Part I, and near visual acuity was measured using the LogMAR word reading cards at the participant’s preferred reading distance. The best-corrected distance visual acuity (BCDVA) was measured after objective refraction by an autorefractor (Nidek ARK900; Nidek Inc., Aichi, Japan). For each eye, IOP by a non-contact tonometry, axial length, K1 (keratometry for flat meridian), K2 (keratometry for steep meridian), and ACD by an IOL-master (Carl Zeiss Meditec, Jena, Germany) were examined at least three times, then the average readings were recorded.

The B-mode ultrasound (10 MHz or 20 MHz, Cine-Scan, Quantel, France) and OCT (Spectralis OCT, Heidelberg Engineering, Heidelberg, Germany) examinations (after 20 minutes’ dark adaptation with the pupil naturally dilated or adequate pupil dilatation with Mydrin-P) were conducted on all 68 participants by two ophthalmologists, respectively, who were trained and certified by retinal specialists. Posterior staphyloma and the kinetic movements of both the posterior vitreous and the vitreoretinal traction were observed under biomicroscopy, ophthalmoscopy and B-mode ultrasonography. The PVD before the macular region was confirmed when a complete separation of the posterior hyaloid membrane (a floating continuous thin membrane-like echo in the vitreous cavity under ultrasonography) and an optically or acoustically empty subhyaloid space were both present under ultrasonography, and no vitreoretinal adhesion at the macular region was present under OCT examination.

OCT examinations, including detected iERM in OCT images, were performed in both groups [38]. The retinal thickness of the central fovea, the thickness of iERM, and the distance between the membrane and central fovea were measured. Most of the OCT scans of the macula were centered on the participant’s fixation point. When visual acuity in the participant’s eye to be scanned was too poor to provide stable fixation, manual positioning of the macula by moving the fixation LED or using external fixation was used. Part of the optic disc was included at the edge of the images to help orient the images.

### Data Management and Analysis

Statistical analyses were performed with SPSS statistical software version 13.0 (SPSS Inc., Chicago, IL, USA). An alpha level of P<0.05 was chosen as the criterion for significance.

Descriptive statistical analyses were performed to characterize demographic data, visual acuity, and clinical characteristics. Age-standardized prevalence was calculated by direct methods using 2000 Chinese national census population. Logistic regression was employed to determine the independence of potential risk factors for iERM, including continuous (age and BMI) and dichotomous variables (gender, level of education, hypertension, diabetes, cardio-cerebrovascular diseases, and high myopia). Odds ratios (ORs) and 95% CIs were reported. Moreover, the independent-samples t-test and Mantel-Haenszel chi-square test were used to determine the significant differences between the case and control groups.

## Results

A total of 4,153 residents were determined as eligible, and 3727 residents underwent interviews and clinic examinations, corresponding to a response rate of 89.7%. Of these, gradable retinal photographs for epiretinal membranes were available for 3571 participants (95.8%, 7,142 eyes; 1,989 women). The mean age was 71.08±7.36 years (median, 71 years; range, 60–98 years). The other 156 participants (4.2%) had un-gradable retinal photographs due to refractive media opacity, such as cataract and vitreous opacity. 245 of 3571 participants (6.9%) were excluded because of a known secondary cause of the development of ERM. DR was present in 32 participants, retinal vascular disease in 50, a history or signs of retinal detachment in 19, and a history of cataract surgery in 162.

**Figure 1:**
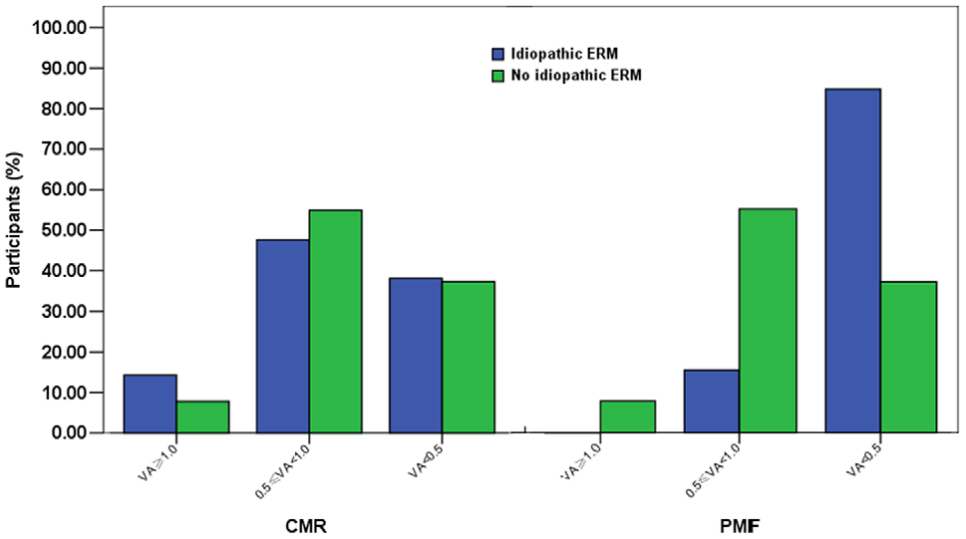
LogMAR presenting visual acuity of idiopathic epiretinal membranes (iERM) and no iERM.

Of the resulting 3,326 participants, iERM was detected in 39 eyes of 34 participants according to retinal photographs, for a prevalence of 1.02%. CMR was present in 0.63% (21 eyes of 21 participants) and PMF in 0.39% (14 eyes of 13 participants). The age-specific, gender-specific, and age-standardized (according to the 2000 Chinese national census population aged 60 years or older) prevalence of CMR, PMF and any iERM are listed in Table 1. Participants’ demographic and clinical characteristics are shown in Table 2. There were significant differences between the participants with and without iERM in level of education and prevalence of diabetes (P<0.05). Compared with the participants without iERM, those with iERM had decreased presenting visual acuity, which was assessed in the worst eye, and a significant difference was observed (P<0.05). Moreover, presenting visual acuity was significantly worse in eyes of the participants with PMF than without iERM (P<0.01), but the participants with CMR had similar presenting visual acuity to those without iERM (Figure 1). After excluding participants with any known secondary cause for the development of ERM (n = 245), the prevalence of iERM was significantly associated with diabetes (OR: 2.457; 95% CI: 1.137, 5.309) and higher level of education (OR: 1.48; 95% CI: 1.123, 1.952). iERM was not associated with age, gender, BMI, hypertension, cardio-cerebrovascular diseases, or high myopia.

**Table 1:** Prevalence of idiopathic epiretinal membranes by age and gender.

Variable		Total (n)	CMR (n, %)	PMF (n, %)	Any iERM (n, %)
Sex*	Male	1481	12 (0.8)	5 (0.3)	17 (1.1)
	Female	1845	9 (0.5)	8 (0.4)	17 (0.9)
Age (y)	60–69	1409	8 (0.6)	6 (0.4)	14 (1.0)
	70–79	1507	11 (0.7)	7 (0.5)	18 (1.2)
	80+	410	2 (0.5)	0 (0)	2 (0.5)
Total*		3326	21 (0.6)	13 (0.6)	34 (1.0)

CMR, cellophane macular reflex; PMF, preretinal macular fibrosis; iERM, idiopathic epiretinal membrane.

* Age-standardized prevalence using the 2000 Chinese national census.

**Table 2:** Demographic and clinical characteristics among the participants (n = 3326) with or without idiopathic epiretinal membrane.*

Characteristic	iERM	No iERM	Statistic value †	P value
Participants [No. (%)]	34 (1.02)	3292 (98.98)		
Mean age (SD, 95% CI), years	71.53 (6.11, 95% CI,69.40 to 73.66)	70.84 (7.34, 95% CI,70.59 to 71.09)	t = 0.568	0.57
60–69 [No. (%)]	14 (41.2)	1395 (42.4)	χ2 = 1.356	0.544
70–79 [No. (%)]	18 (52.9)	1489 (45.2)		
80+ [No. (%)]	2 (5.9)	408 (12.4)		
Male [No. (%)]	17 (50.0)	1464 (44.5)	χ2 = 0.416	0.519
Mean BMI (SD, 95%CI)	24.15 (3.02, 95% CI,23.10 to 25.20)	23.90 (3.27, 95% CI,23.79 to 24.02 )	t = 0.436	0.663
Mean education (SD, 95%CI) years	9.38 (5.38, 95% CI, 7.51 to 11.26 )	7.42 (4.47, 95% CI, 7.27 to 7.58 )	t = 2.54	0.011 †
Illiterate [No. (%)]	4 (11.8)	468 (14.2)	χ2 = 10.725	0.023 †
Primary school [No. (%)]	6 (17.6)	1143 (34.7)		
Junior high school [No. (%)]	9 (26.5)	814 (24.7)		
Senior high school [No. (%)]	6 (17.6)	551 (16.7)		
College or higher [No. (%)]	9 (26.5)	361 (9.6)		
Systemic comorbidities suffered				
Hypertension [No. (%)]	18 (52.9)	1310 (39.8)	χ2 = 2.425	0.119
Diabetes [No. (%)]	9 (26.5)	435 (13.2)		0.038 ‡
Cardio-cerebrovascular diseases [No. (%)]	6 (17.6)	292 (8.9)		0.118
Hypermyopia [No. (%)]	0 (0)	163 (5.0)		0.411
Mean logMAR presenting VA (SD, 95%CI)	0.44 (0.29, 95% CI, 0.34 to 0.55 )	0.54 (0.25, 95% CI, 0.53 to 0.55)	t = −2.263	0.024 ‡
Mean logMAR UCDVA (SD, 95%CI)	0.39 (0.29, 95% CI, 0.29 to 0.50)	0.45 (0.27, 95% CI, 0.44 to 0.46)	t = −1.205	0.228

iERM, idiopathic epiretinal membrane; SD, standard deviation; CI, confidence interval; BMI, body mass index; VA, visual acuity; UCDVA, uncorrected distance visual acuity.

* Idiopathic epiretinal membrane was considered present in participants without a secondary cause (diabetic retinopathy, retinal vascular disease, retinal detachment, or history of cataract surgery) of ERM.

† t: Independent samples t-test; χ2: Pearson chi-square.

‡ P<0.05.

In the case-control study, the demographic characteristics of the 34 participants with iERM and the 34 healthy participants were compared in Table 3. The difference between the two groups was not statistically significant in age, gender, BMI, diabetes history, or level of education. In contrast to serum total cholesterol (t = 2.47, p = 0.02), the difference between the two groups was not statistically significant in fasting plasma glucose, serum creatinine, or triglyceride (P>0.05). The fasting plasma glucose levels of the iERM group(mean 6.25 mmol/L, SD 1.79) and control group (mean 6.12 mmol/L, SD1.8 ) were both slightly higher than the normal range (3.9–6.10 mmol/L), and serum total cholesterol was higher in the control group (mean 5.53 mmol/L, SD 1.17; normal range <5.20 mmol/L). In contrast to distance visual acuity (t = −2.25, P = 0.03) and near visual acuity (t = −2.32, P = 0.02), the differences in ocular biological parameters, including refractive error, axial length, K1, K2, ACD and IOP, between the two groups were not statistically significant (P>0.05). When we compared the distance visual acuity of the participants with CMR or PMF, respectively, with the controls, the distance visual acuity was significantly lower in the eyes with PMF (p<0.01), while it was similar between CMR and the controls. Twelve eyes of 9 participants (26.5%) with iERM were associated with PVD before the macular region, while 3 participants (8.8%) were associated with PVD in the control group, but the differences between the two groups were not statistically significant (P = 0.056). None of the eyes had posterior staphyloma. According to OCT images, there was a significant difference in the mean retinal thickness of the central fovea (P<0.01) between the iERM group (390.78 µm, SD 128.60) and control group (243.55 µm, SD 25.33). Moreover, the mean thickness of iERM was 20.03 µm (SD 13.04), and the mean distance between the membrane and central fovea was 65.76 µm (SD 225.99).

**Table 3:** Demographic characteristics in the 34 participants with iERM and the 34 healthy participants (control group).

	iERM group	Control group	Statistic value*	P value
No. of participants	34	34		
Mean age (SD) years	72.53 (6.11)	70.44 (7.90)	t = 1.219	0.227
Male [No. (%)]	17 (50.0)	15 (44.1)	χ2 = 0.236	0.627
Mean BMI (SD)	24.15 (3.02)	23.02 (3.54)	t = 1.412	0.163
Levels of education				
Illiterate [No. (%)]	4 (11.8)	4 (12.5)	χ2 = 1.59	0.87
Primary school [No. (%)]	6 (17.6)	3 (9.4)		
Junior high school [No. (%)]	9 (26.5)	10 (31.3)		
Senior high school [No. (%)]	6 (17.6)	7 (21.9)		
College or higher [No. (%)]	9 (26.5)	8 (25)		
Diabetes suffered [No. (%)]	9 (26.5)	4 (11.8)	χ2 = 2.378	0.123

iERM, idiopathic epiretinal membrane; SD, standard deviation.

* χ2: Mantel-Haenszel chi-square; t: independent-samples t-test.

## Discussion

In this population-based study, we observed an overall iERM prevalence of 1.02% in Beixinjing Blocks, Shanghai, China, which included 0.63% for CMR and 0.39% for PMF. Our study suggests that iERM is less frequent in urban Chinese than reported in samples of Asians from the Handan Eye Study (3.0%) [25] and Singapore Malay Eye Study (9.5%) [2], Caucasians from the Melbourne Visual Impairment Project Study (5.4%) [23] and Latinos from the LALES (17.5%) [8]. Therefore, the prevalence of iERM differs among population-based studies, for reasons unknown. One possible reason may be ethnic differences, as mentioned by some previous studies [22], [24]. Interestingly, not just the prevalence of iERM but the prevalence of DR and age-related macular degeneration in our previous studies [39], [40] were lower than in Western countries. Another possible reason is different inclusion criteria for eligible participants. In the present study, urban residents aged 60 years or older were randomly selected, while most of the other studies used an inclusion criterion of 40 years or older [8], [23], [26]. The prevalence of diabetes [4], [8], [27], a risk factor for iERM, was 20.4% among persons who were aged≥60 years in China [41], and Shanghai, as the largest city and one of the most economically developed areas, has a higher prevalence (aged 60–69 years: 22.4%/male, 22.3%/female; aged 70–74 years: 25.6%/male, 27.2%/female) [42], which was close to the results from the Singapore Malay Eye Study (21.8%) [43], but much lower than the LALES (34.5%) [44]. Therefore, we cannot rule out the possible association between the prevalence of diabetes and the lower prevalence of iERM in Beixinjing Blocks. In addition, cataract surgical rate (CSR) in Beixinjing Blocks (aged≥60 years) was 7,790/million in 2007 [45]. Approximately 8000 cataract surgeries (an exclusion criteria for iERM) per year have been closer to the western developed countries, which might cause lower prevalence of iERM in Beixinjing Blocks. Nevertheless, some methodological issues should be mentioned. This study used non-stereoscopic 45° retinal photographs to identify and grade iERM, whereas some other studies used 30° stereoscopic retinal photographs and/or OCT [8], [23]–[25]. Even though we trained ophthalmologists to evaluate the participants for iERM, non-stereoscopic retinal photographs might have resulted in an underestimation of the prevalence of iERM by missing subtle early macular changes, especially CMR.

Consistent with previous studies [4], [8], [27], our study found that diabetes was positively associated with the prevalence of iERM. Samantha and associates [8] speculated that the high prevalence of iERM (17.5%) in their population-based study was because of its high prevalence of diabetes. These findings suggest diabetes might promote the occurrence and development of iERM. A conceivable pathological mechanism is that synchysis contributes to the precocious and exaggerated PVD in diabetics, and therefore, PVD is significantly more common in diabetics, even in the absence of retinopathy [46].

In addition to diabetes, we found that a higher level of education was associated with iERM, which was consistent with the Beijing Eye Study [24]. In contrast to previous studies, we failed to find a significant association between the prevalence of iERM and other potential risk factors, including older age [4], [7], [8], [22]–[25], [26], [28], gender [26], and high myopia [4], [8]. It was likely that the number of participants with iERM was too small in our study to detect associations with these factors. Not surprisingly, we found that presenting visual acuity was significantly worse in eyes of participants with iERM or PMF, but not in those with CMR, compared with participants without iERM. These findings are consistent with previous studies [4], [7], [25]. The presence of PMF alone can cause decreased visual acuity if it involves the center of the fovea [4], [7], [8]. It was conceivable that most iERM cases detected from retinal photographs or OCT were early-stage iERM, so most patients with iERM had no obvious visual impairment.

In the subsequent case-control study, we unexpectedly found that serum total cholesterol was negatively associated with iERM. However, hypercholesterolemia has been reported as a possible risk factor for iERM in the Hisayama Study [22] and the Multi-Ethnic Study of Atherosclerosis [47]. Although the pathophysiological mechanisms of the formation of iERM are not clear, experimental studies demonstrate that chemoattractants from the serum or vascular endothelial cells may mediate cell migration and proliferation, which might promote the development of iERMs in patients with hyperlipidemia [48], [49]. Therefore, we speculated that the cholesterol association was a spurious finding in our study, due to the small sample and possible sampling error.

There is controversy [8], [23]–[26] about the relationship between refractive error and iERM, especially myopia [23], [25], [28], which might have a positive association with iERM. However, in addition to distance visual acuity and near visual acuity, no ocular biological parameters were significantly different between the two groups in our study. It was notable that the incidence of PVD in the case group was much higher than in the control group, although this difference was not statistically significant. Large clinical studies [32]–[34], [50] have implicated PVD as a factor involved in the genesis of iERM [15]. Therefore, we cannot rule out the possibility that PVD has clinical significance in iERM.

The limitations of our study should be stated. First, blood biochemical parameters, such as serum total cholesterol [22] and fasting plasma glucose [4], that were previously reported as risk factors for iERM were not examined in our population-based study due to the limited resources. Second, it is difficult to complete B-mode ultrasound, OCT, and IOL-master examinations for all participants in large-scale population-based studies, such as the Handan Eye Study [25], in which only 85.3% participants had OCT images from at least one eye that were considered gradable for ERM. Although we performed a further case-control study, residual confounding was also possible. In addition, the diagnosis and grading of iERM could be affected by non-stereoscopic retinal photographs and refractive media opacity, such as cataract and vitreous opacity, which may have led to an underestimation of the prevalence of iERM.

In conclusion, iERM occurs at a relatively low frequency in a population-based sample of Beixinjing Blocks aged 60 years or older. Its prevalence was lower than in Western countries and in Chinese subjects in Handan, and it was associated with diabetes and higher level of education. Furthermore, iERM causes a substantial decrease in visual acuity.
